# Working memory exertion after simultaneous interpreting in bilinguals

**DOI:** 10.1017/S1366728925100898

**Published:** 2026-01-02

**Authors:** Isabelle Chou, Agustina Birba, Jiehui Hu, Edinson Muñoz, Guoqing Kwon, Adolfo M. García

**Affiliations:** 1 Sichuan University College of Foreign Languages and Cultures, China; 2 Cognitive Neuroscience Center (CNC), University of San Andres, Argentina; 3 Instituto Universitario de Neurociencia, Universidad de La Laguna, Spain; 4 Facultad de Psicología y Logopedia, Universidad de La Laguna, Spain; 5 School of Foreign Languages, University of Electronic Science and Technology of China, China; 6 Departamento de Lingüística y Literatura, Universidad de Santiago de Chile Facultad de Humanidades, Chile; 7 Global Brain Health Institute (GBHI), University of California, San Francisco, CA, United States & Trinity College Dublin, College Green, Dublin, Ireland

**Keywords:** bilingualism, simultaneous interpreting, working memory, cognitive exertion

## Abstract

Simultaneous interpreting (SI), a challenging task enabled by bilingualism, is claimed to distinctly tax working memory (WM). However, causal designs are missing, limiting our understanding of the phenomenon. We recruited 50 Chinese-English bilinguals and assessed their WM performance (alongside inhibitory and fluency outcomes) before and after L1–L2 SI or a control task (text comprehension). WM scores (especially under high-demand, multimodal conditions) increased after text comprehension but not after SI, adjusting for age of L2 appropriation, years of L2 use, L2 proficiency and SI competence. Of note, WM was assessed immediately before and after SI, ruling out other cognitive influences. Conversely, no distinct patterns were observed on inhibitory or fluency tasks. Briefly, this activity seems to hinder practice-related WM gains – a finding that expands contemporary accounts of interpreting.

## Highlights

- We examined the impact of simultaneous interpreting (SI) on working memory (WM).
- Unlike a single-language task, SI prevented practice-driven WM boosts.
- This effect emerged for multimodal input, adjusting for bilingual experience factors.
- SI did not have a distinct effect on inhibitory or fluency outcomes.
- Such findings inform cognitive models of bilingualism and SI training guidelines.

## 1. Introduction

A distinct trait of bilingualism is the possibility to engage in simultaneous interpreting (SI), the immediate rendition of oral messages from one language into another (García et al., 2019). This activity is proposed to greatly tax working memory (WM), but causal designs are wanting, especially including other tasks and cognitive functions (Timarová, 2008). A fertile ground is thus laid to examine the real-time impact of specific bilingual skills on domain-general systems. Here, we tackle this gap via a cognitive exertion paradigm, comparing WM (and other executive) outcomes before and after SI, relative to a control language processing task.

SI ranks among the most demanding cognitive activities enabled by bilingualism (García, 2014; García et al., 2019; Hervais-Adelman et al., 2015). It requires perceiving, accessing and recalling source-language information to reformulate it in a different language as new input segments are processed (Ahrens, 2017; Chernov, 2004; Gerver, 1976; Paradis, 1994, 2009). Such operations occur iteratively, with input typically delivered at >120 words per minute (Chernov, 2004; Gerver, 1975), ear-voice spans lasting 2–4 seconds (Anderson, 1994; Gerver, 1976) and input–output overlaps accounting for 70% of total processing time (Chernov, 1994). These challenges are such that, to prevent task disengagement, reduced output quality and early burnout, professionals are recommended not to perform SI for more than 30 minutes at a time (AAIC, 2025; García, 2019; Moser-Mercer et al., 1998).

Unsurprisingly, the activity entails diverse cognitive costs. SI increases stress responses such as cortisol (Moser-Mercer et al., 1998) and heart rate (Rojo López et al., 2021), especially under heightened time pressure (Korpal, 2016). Also, relative to single-language tasks, like text listening and shadowing, SI involves greater self-reported mental workload (Boos, 2022), objective cognitive exertion markers, including increased pupil dilation (Hyönä et al., 1995; Seeber & Kerzel, 2011), and activation along cortico-subcortical executive brain regions (Hervais-Adelman et al., 2011, 2015). As advanced by several models (Gile, 1999; Mizuno, 2005; Seeber, 2011), SI would place distinct demands on WM, a system supporting the transient storage and combination of information as other processes unfold (Hervais-Adelman & Babcock, 2020).

Indeed, SI entails increased pupil dilation when processing WM-taxing stimuli – for example, asymmetrical structures such as verb-final constructions, as found in Japanese or German (Seeber & Kerzel, 2011). Also, relative to single-language tasks, SI involves higher theta power (a neural marker of WM demands) (Boos, 2022) and prefrontal activation peaks during input–output overlaps (i.e., instances that maximally tax WM) (Hervais-Adelman et al., 2015). Moreover, SI quality correlates with WM outcomes (Bae & Jeong, 2021; Injoque-Ricle et al., 2015; Mellinger & Hanson, 2019), and sustained practice is consistently linked to advantages in this domain (as SI continuously requires *recalling and integrating* input while concurrent processes are deployed) (Chernov, 2004). Such demands seem to peak in the L1–L2 direction, which, relative to the L2–L1 direction, involves greater modulation of WM-relevant brain mechanisms, such as inferior frontal activation (García, 2019; Rinne et al., 2000; Tommola et al., 2000), temporo-frontal connectivity (Zheng, et al., 2020) and frontal theta oscillations (Pérez et al., 2022).

Of note, WM taxation during SI may depend on cognitive demands. Unlike untrained multilinguals, interpreters show training effects in highly taxing (multimodal *n*-back) WM tasks but not in simpler (single-domain) tasks (Morales et al., 2015). Suggestively, no systematic expertise-related advantages are observed in less critically taxed domains, like inhibition (as both languages need to be simultaneously active rather than alternatively suppressed) and verbal fluency (since SI does not require producing arbitrary sequences of decontextualized words) (García et al., 2019).

SI, then, would distinctly tax WM. However, evidence for this claim remains limited. Relevant studies employ cognitively ambiguous markers (e.g., pupil dilation) (Seeber & Kerzel, 2011), WM tests done in the absence of actual SI (Boos, 2022; García et al., 2019) or task-related neural measures failing to isolate or manipulate WM (Boos, 2022; Hervais-Adelman et al., 2015). Moreover, *causal* evidence is scant and limited to comparisons of outcomes before and after months-long training programs. SI practice seems to boost WM performance (Ünlü & Şimşek, 2018) and to lower the engagement of WM-relevant brain regions during the task (Hervais-Adelman et al., 2015), suggesting that this domain is systematically recruited by practicing interpreters. Conversely, other cognitive functions, such as inhibitory skills (Van de Putte et al., 2018), do not exhibit changes before and after SI training, indicating that they are less systematically recruited by this activity. Overall, the real-time impact of SI on WM remains poorly understood, precluding insights into how particular domain-general skills are recruited during specific bilingual processes.

Such gaps can be tackled via cognitive exertion paradigms. In these paradigms, specific domains are assessed before and after a given task, on the assumption that those more critically engaged will be drained and hence impaired upon post-task testing. This approach has revealed critical links between particular tasks and circumscribed cognitive functions, by revealing exertion of inhibitory capacity during emotional suppression (Wessel et al., 2010), learning capacity during extended periods of instruction (Chen et al., 2018), self-regulatory resources during high attentional exertion (Converse & DeShon, 2009) and positive emotion mechanisms during overly enthusiastic behavior (Maranges et al., 2017). More directly, a study on phonological interference effects during SI found that number recall dropped when digit lists were simultaneously translated (as opposed to simply heard or repeated in the same language) before being uttered (Darò & Fabbro, 1994). Similar designs could reveal the causal impact of SI on WM, as the need to transiently store, recall and integrate input information during output production would exhaust relevant mnesic mechanisms and render them suboptimally available for further WM tasks.

Against this background, we conducted a cognitive exertion study on SI. We asked SI trainees to perform a validated WM task (involving unimodal and multimodal stimuli) before and after (a) a 10-minute-long L1–L2 SI session or (b) a control text comprehension (TC) task. To test for specificity, participants also completed tasks tapping other SI-relevant domains (viz., inhibitory control and context-free vocabulary navigation, via Stroop and verbal fluency tasks). We hypothesized that L1–L2 SI would distinctly reduce WM scores, as opposed to less critical mechanisms, such as inhibitory and fluency resources. We further predicted that such an effect would be specific to SI, compared to TC. Finally, we anticipated that the effect would be uninfluenced by inter-individual variability in bilingualism-related variables (age of L2 appropriation,^1^ years of use of L2, L2 proficiency, SI competence). Strategically, the use of a high-demand (multimodal integration–recall) and a low-demand (unimodal recall-only) condition allowed testing whether predicted effects were effort-dependent. With this approach, we aim to inform models linking bilingual abilities to specific executive domains.

## 2. Method

### 2.1. Participants

**Figure 1.**
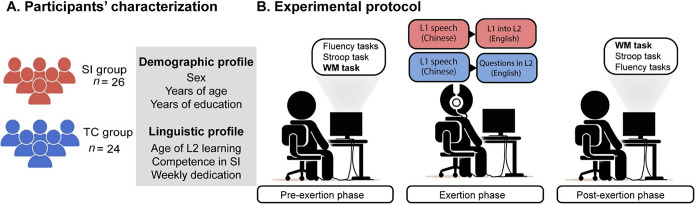
Study design. (A) Fifty Chinese-English bilinguals were randomly assigned to the SI group (*n* = 26) and to the TC group (*n* = 24), both matched for demographic and linguistic variables. (B) The experiment involved a pre-exertion phase (including the WM assessment and control tasks), an exertion phase (involving an SI task for the SI group and a TC task for the TC group) and a post-exertion phase (featuring the same initial tasks in reverse order). SI = simultaneous interpreting, TC = text comprehension.

We recruited 55 right-handed Chinese-English bilinguals enrolled in the Master’s in Translation and Interpreting at the University of Electronic Science and Technology of China (UESTC). They all had normal vision and hearing and reported no neurological impairment. Participation was compensated with course credits. Participants were randomly assigned to the L1–L2 SI or the TC task. Upon removal of five participants due to poor task compliance or withdrawal, the final sample of 50 (Figure 1A) involved 26 SI participants and 24 TC participants – reaching a power of 1 (Supplementary Material 1). Demographic and language profile data for group matching were collected via the Translation and Interpreting Competence Questionnaire (TICQ), a validated (Schaeffer et al., 2020) and previously reported (Chou et al., 2021; Jacob et al., 2024) online self-rating instrument gathering quantitative and qualitative data on demographics, language history and translation/interpreting competence. To favor between-group matching, SI and TC participants were pooled from the same master’s courses. TICQ data showed that both groups were matched for sex, age and education, as well as age of L2 appropriation, years of L2 use, L2 proficiency, SI competence and weekly dedication to SI (Table 1). Importantly, all participants showed upper-intermediate levels of L2 proficiency on the TICQ (on a scale of 0–100), consistent with the incidental observation that they each possessed at least one language proficiency certification (Test for English Majors, level 4 or higher; the China Accreditation Test for Translators and Interpreters, level 3; or the International English Language Testing System). All participants signed written informed consent. The study protocol was approved by the institutional ethics committee and adhered to the Declaration of Helsinki.

**Table 1.** Participants’ demographic and linguistic profiles

Demographic profile	SI group	TC group	χ^2^/*F*	*p*-value
Sex (F:M)	22:4	21:3	Χ^2^ (1) = .00	.99
Years of age	23.89 (3.57)	23.42 (1.10)	*F* (1,48) = .38	.53
Years of education	17 (2.44)	18.0 (1.44)	*F*(1,48) = 3.14	.08
**Linguistic profile**				
Age of L2 appropriation	8.56 (2.71)	8.58 (2.00)	*F*(1,48) = .0017	.96
Years of L2 use	12.1 (4.70)	11.9 (4.62)	*F*(1,48) = .0082	.86
Global L2 proficiency	70.4 (10.2)	73.1 (12.7)	*F*(1,48) = .59	.55
L2 speaking proficiency	66 (9.75)	70.4 (11.2)	*F*(1,48) = 2.26	.14
L2 reading proficiency	76.7 (13.7)	79.4. (14.5)	*F*(1,48) = .47	.50
L2 listening proficiency	69.1 (18.8)	69.3 (14.7)	*F*(1,48) = .0012	.97
L2 writing proficiency	68.3 (11.8)	71.1 (12.7)	*F*(1,48) = .066	.42
L1–L2 SI competence	59.70 (15.44)	66.29 (10.90)	*F*(1,48) = 3.02	.08
L2–L1 SI competence	60.63 (16.89)	67.79 (10.50)	*F*(1,48) = 3.20	.08
Weekly dedication to L1–L2 SI	2.81 (2.83)	5.17 (4.83)	*F*(1,48) = 3.72	.07
Weekly dedication to L2–L1 SI	3.48 (3.35)	4.83 (4.33)	*F*(1,48) = 0.15	.70

*Note*: Data are shown as mean (*SD*). Statistical comparisons were performed via chi-squared test for sex and via ANOVA for every other variable. SI = simultaneous interpreting, TC = text comprehension.

### 2.2. Study design

The study consisted of three phases (Figure 1B). First, in the pre-exertion phase, participants completed three control tasks (Stroop, semantic fluency, phonemic fluency) and then performed the WM task. Second, in the exertion phase, the SI group performed an L1–L2 SI task, while the TC group completed a TC task. Finally, in the post-exertion phase, participants completed the same tasks of the pre-exertion phase, in reverse order. Across phases, participants sat at a desk in a dimly lit room, facing a desktop computer, with no external distractions. All instructions were delivered orally in L1 (Chinese). Overall, the experiment lasted roughly 2 hours.

#### 2.2.1. Pre-exertion phase

**Figure 2.**
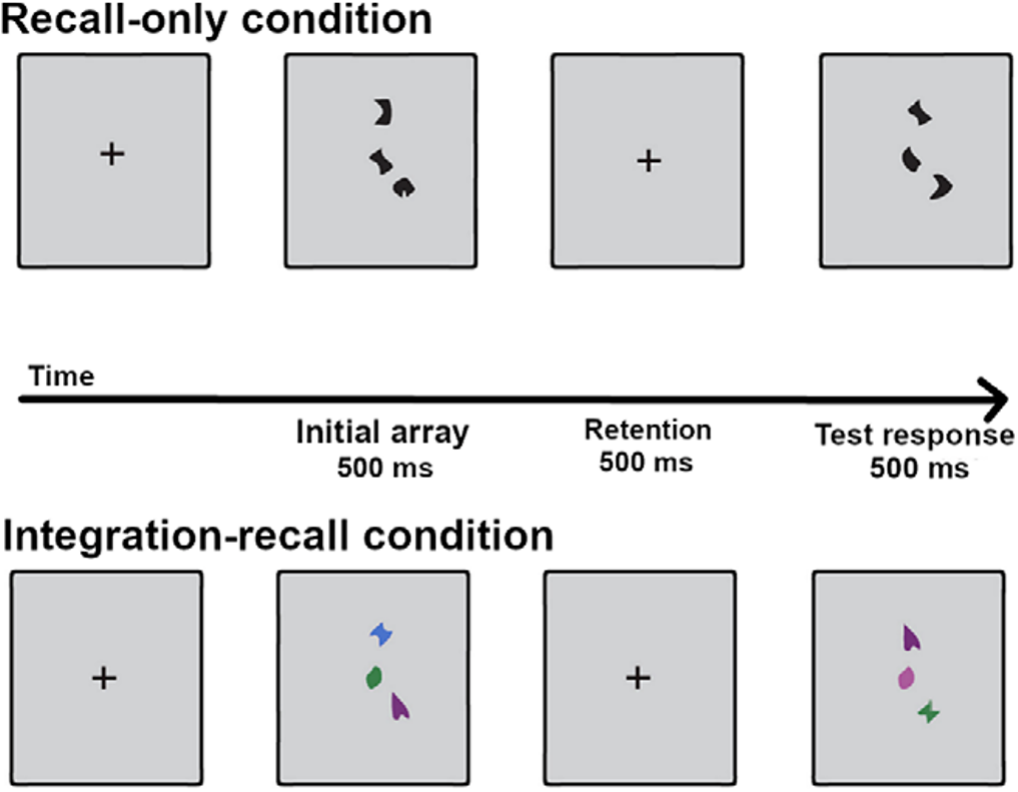
Trial structure in the WM task. The task involves an initial array of items, followed by a retention period and then a test array. Stimuli in the test array may or may not match those of the initial array. The top row illustrates the ‘recall-only’ condition, tapping on unimodal WM retrieval by manipulating black shapes. The bottom row exemplifies the ‘integration–recall’ condition, assessing multimodal WM retrieval by manipulating both the shapes and their color.

*Working memory task.* WM was assessed via a widely validated task (Baddeley et al., 2011; Birba et al., 2017; Brockmole et al., 2008) displaying stimulus arrays on a computer screen (Figure 2). Validated in diverse populations (Brockmole et al., 2008; Parra et al., 2009, 2014), this task involves high WM exertion under multimodal integration conditions. Importantly, its non-verbal nature circumvents language-proficiency confounds and priming effects triggered by the SI text. The task included two conditions, tapping on (a) recall and (b) integration–recall skills. In the ‘recall-only’ condition, participants viewed initial arrays of black shapes and, after a delay, indicated whether a test array featured the same shapes. In the ‘integration–recall condition’, initial shapes were presented in different colors and, after a delay, participants indicated whether the test array presented the same shape–color combinations. A total of 8 six-sided random polygons and eight colors were utilized for the tasks. Stimuli were presented with a standard 17-inch PC monitor with a resolution of 1920*1080 pixels, each occupying 1° of visual angle and positioned within a 10° area. Participants were seated at a viewing distance of 70 cm from the screen.

Each trial commenced with a fixation cross displayed for 500 ms, followed by a random interval lasting between 400 and 600 ms. Initial three-item arrays were then presented for 500 ms, during which participants had to memorize the items. This was followed by a 900-ms retention interval, with no stimuli. A three-item test array appeared next for 500 ms, and participants were required to determine whether these stimuli matched those in the initial display by pressing ‘1’ for ‘match’ or ‘2’ for ‘non-match’ (with key assignments counterbalanced across trials). The next trial began only after participants made a response. Half the trials were ‘match trials’ (with initial and test trials being identical), while the other half were ‘non-match trials’ (with different initial and test trials).

In the ‘recall-only’ condition, ‘non-match trials’ were created by replacing two black shapes from the initial array with new black test shapes, requiring participants to rely solely on shape encoding to detect changes. In the ‘integration–recall’ condition, ‘non-match trials’ were created by swapping the colors of two initial items, requiring participants to integrate shape and color information to detect the change. No shape or color was repeated within a single array. Conditions were counterbalanced and trial order was randomized across participants. The task lasts roughly 20 minutes.

*Inhibitory control task.* Inhibitory control was evaluated via a color–word Stroop task, amply validated in diverse populations, bilinguals with SI experience (Aparicio et al., 2017; MacPherson, 2019; Peng et al., 2017). Facing a computer screen, participants were shown color words (‘red’, ‘yellow’, ‘blue’, ‘green’) typed in different ink colors, and they had to indicate the name of the ink as quickly as possible by pressing a given key (‘f’ for red, ‘j’ for yellow, ‘v’ for blue, ‘n’ for green). The task included 16 combinations of words and ink colors, used to create 40 congruent trials (color words written in their corresponding color) and 60 incongruent trials (color words written in a different color), with each congruent combination shown ten times and each incongruent combination shown five times.

Each trial started with a fixation cross displayed for 500 ms, followed by a stimulus word presented for 1,500 ms, during which participants had to respond. A random interval between 500 and 1,000 ms preceded the following trial. All words were presented in the participants’ L1. Trials were randomly distributed across congruent and incongruent categories. In the pre-exertion phase, the task began with a practice block, featuring four congruent and 12 incongruent trials. Stimulus administration and response recording were handled via E-Prime 3.0 (Psychology Software Tools, Pittsburgh, PA). Altogether, the task lasted roughly 4 minutes.

*Context-free vocabulary navigation tasks.* Context-free vocabulary navigation skills were evaluated via one-minute verbal fluency tasks, previously reported in SI research (Dottori et al., 2020; Santilli et al., 2019). The semantic fluency task had participants name as many exemplars of a category as possible, for 60 seconds, first in their L1 and then in their L2. To mitigate practice and priming effects, we counterbalanced the categories ‘animals’ and ‘fruits’ between the pre- and post-exertion phases. Within each phase, participants were presented with the same category for both Chinese and English. As to the phonemic fluency task, participants were asked to generate as many words as possible beginning with specific phonemes, orally in English and in written form in Chinese. The order of languages was counterbalanced between the pre- and post-exertion phases, as were the phonemes used for English (/f/, /s/) and for Chinese (/k/, /t/). Following previous studies, we adopted the written modality for Chinese to circumvent issues related to tonal variations of specific characters (e.g., /ma/ means ‘mother’ with tone 1, ‘bother’ with tone 2, ‘horse’ with tone 3, ‘curse’ with tone 4) (Eng et al., 2019). All responses were audio-recorded in .mp3 format, transcribed by one examiner and subsequently verified by a second examiner to ensure accuracy (a third examiner was summoned to settle the very few instances yielding disagreement). Following validated procedures (Nichols et al., 2019; Selnes, 1991), responses were excluded as invalid if they consisted of either proper names, or repetitions, derivations or affixations of previous responses. In each language, semantic fluency is scored by counting the number of correct, unique words produced within the semantic category in the given time frame. Phonemic fluency scores are calculated by counting the number of correct, non-repeated words in the given time frame.

#### 2.2.2. Exertion phase

The exertion phase involved a specific naturalistic language processing task for each group: SI for the SI group (as our target condition) and TC for the TC group (as our control condition). The stimulus for both tasks consisted of the first 10 minutes of former Singapore Prime Minister Lee Hsien Loong’s speech during the 2022 National Day Rally (https://www.pmo.gov.sg/Newsroom/National-Day-Rally-2022-Chinese). The material comprised 1,977 Chinese characters, delivered at a rate of 3.3 characters per second, and it was presented binaurally via a stereo headset after participants adjusted the volume to their preferred level. As in previous works (Bae & Jeong, 2021; Boos et al., 2022; Tzou et al., 2012), no prior information was provided on the topic – a scenario that mirrored participants’ training conditions and is often encountered in professional settings (Chang et al., 2018). Note-taking with pen and paper was allowed during the task.

The SI group was instructed to listen to the speech and simultaneously interpret it into English, preserving as much information as possible while producing idiomatic L2 renditions. The use of the L1–L2 direction was strategic to enhance ecological validity, as (i) it is predominant in professional SI across China and (ii) participants were more acquainted with it than with L2–L1 SI in their training program. Output was recorded through the headset’s built-in microphone and saved as .mp3 files on Xima 3100 Digital Integrated Teaching System V2.0. SI quality was evaluated according to standard criteria, focusing on content, form and delivery (Hamidi & Pöchhacker, 2007; Macías, 2006; Pöchhacker, 2001). The TC group was asked to listen attentively so as to respond to five open-ended questions presented in a printed survey at the end of the recording. Questions were formulated in L2 (English) and answered in writing. The use of L2 for this task increased task demands (creating more stringent conditions to test the selectivity of predicted SI-related exertion effects), while favoring comparability with the SI task by mirroring its dual-language nature.

#### 2.2.3. Post-exertion phase

The post-exertion phase was identical to the pre-exertion phase, except that tasks were presented in reverse order, with the WM task first, followed by the Stroop and fluency tasks. Through this sequencing, the WM task was always performed immediately before and immediately after the corresponding exertion task, preventing interference from other tests.

### 2.3. Statistical analysis

Across tasks, accuracy and response time analyses were performed upon removing outlier values at ±2.5 standard deviations from the corresponding group’s mean. First, WM task accuracy was analyzed using linear mixed-effects models separately for each ‘condition’ (recall-only, integration–recall), with ‘phase’ (pre-exertion, post-depletion) and ‘group’ (SI, TC) as fixed effects, with a random intercept for subjects. To examine whether group differences were driven by variability in WM performance, we first conducted Levene’s tests comparing variances across groups (SI versus TC) and phases (pre-exertion versus post-exertion) for each WM condition (integration–recall and recall-only). In addition, we computed individual change scores (subtraction between the post- and pre-exertion phase outcomes) and visualized their distributions. Group effects on change scores were further assessed using quantile regression at τ = .25, .50 and .75, corresponding to the lower, median and upper parts of the distribution. This approach allowed us to test whether SI selectively influenced participants showing small, typical or large practice-related gains.

Second, for the Stroop task, linear mixed-effects models were separately run on the congruent and incongruent conditions, targeting accuracy and response times (the latter, considering correct responses only). The models included ‘phase’ (pre-exertion, post-exertion) and ‘group’ (SI, TC) as fixed effects and ‘subject’ as a random effect. Additionally, we compared congruent and incongruent trials, for accuracy and response time, using a mixed-effects model with ‘condition’ (congruent, incongruent), ‘phase’ (pre-exertion, post-exertion) and ‘group’ (SI, TC) as fixed effects and ‘subject’ as a random intercept.

Third, fluency tasks’ outcomes were analyzed via linear mixed-effects models, with ‘phase’ (pre-exertion versus post-exertion), ‘language’ (L1 versus L2) and ‘group’ (SI versus TC) as fixed effects, along with their interactions, and a random intercept for ‘subjects’ to account for repeated measures. Across tasks, ANOVA was used to assess the significance of main effects and interactions. To test for robustness despite inter-individual variability in bilingualism-related factors, all analyses were re-run while covarying for age of L2 appropriation, years of L2 use and L2 proficiency (including relevant macroskills), as well as SI competence in the L1–L2 and L2–L1 directions. Post hoc analyses for significant interactions were conducted via Tukey’s HSD tests. Alpha values were set at 0.05. Outlier-adjusted models were also fitted to confirm the robustness of the results. All analyses were performed on R 4.1.1 (Team, 2013).

## 3. Results

### 3.1. Working memory

**Figure 3.**
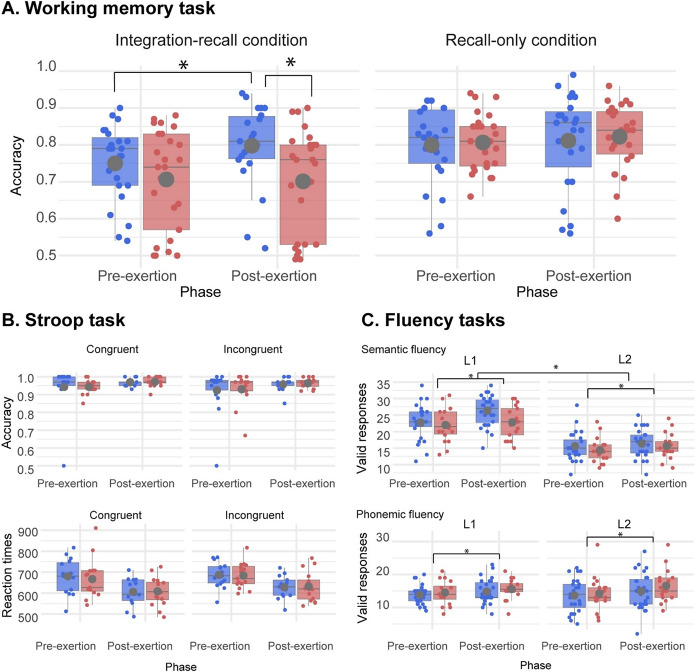
Key results. (A) WM task. Accuracy analyses on the integration–recall condition revealed similar performance between groups in the pre-exertion phase and worse performance for the SI than for the TC group in the post-exertion phase. Also, performance improved between sessions for the TC group but not for the SI group. No significant accuracy effects emerged in the recall-only condition. (B) Inhibitory control task. No significant differences were found between groups or phases for either accuracy or response time. (C) Context-free vocabulary navigation tasks. Semantic (top inset) and phonemic (bottom inset) fluency results revealed significant main effects of phase, with significant increases from pre- to post-exertion. Red and blue dots represent SI and TC group participants, respectively. SI = simultaneous interpreting, TC = text comprehension.

Analysis of accuracy in the integration–recall condition revealed no significant main effects of ‘group’ (*F*_(1,46)_ = 1.89, *p =* .17) or ‘phase’ (*F*_(1,46)_ = .49, *p =* .48), but a significant interaction between ‘phase’ and ‘group’ (*F*_(1,46)_ = 4.8387, *p =* .02) (Figure 3A). Post hoc comparisons (*MSE* = 0.002, *df* = 1, 46) showed that, relative to the TC group, the SI group performed similarly in the pre-exertion phase (*p* = 0.24) and worse in the post-exertion phase (*p* = .02). This interaction remained significant upon controlling for age of L2 appropriation (*F*_(1,44.89)_ = 4.74, *p* = .034), years of L2 use (*F*_(1,44.50)_ = 4.69, *p* = .036), global L2 proficiency (*F*_(1,44.84)_ = 4.75, *p* = .034), L2 listening proficiency (*F*_(1,44.87)_ = 4.76, *p* = .034), L2 speaking proficiency (*F*_(1,44.79)_ = 4.75, *p* = .034), L2 reading proficiency (*F*_(1,44.89)_ = 4.74, *p* = .035), L1–L2 SI competence (*F*_(1,44.86)_ = 4.75, *p* = .035) and L2–L1 SI competence (*F*_(1,44.86)_ = 4.75, *p* = .035). The same was true for the poorer performance of the SI group compared with the TC group in the post-exertion phase (*p* = .02), upon adjusting for age of L2 appropriation, years of L2 use, L1–L2 SI competence and L2–L1 SI competence. Also, integration–recall accuracy improved from the pre- to the post-exertion phase for the TC group (*M*_pre_ = 0.75 [0.10], *M*_post_ = 0.80 [0.11], *p* = .01), but not for the SI group (*M*_pre_ = 0.70 [0.13], *M*_post_ = 0.70 [0.14], *p* = .71). In the integrated model, with ‘condition’ (congruent, incongruent) as a fixed effect, we found a significant main effect of ‘phase’ (*F*_(1,84.68)_ = 8.13, *p* = .005), indicating that participants were more accurate in the second session. There were no other significant effects or interactions, including the three-way interaction (‘condition’ × ‘phase’ × ‘group’: *p* = .98). Response times yielded a significant main effect of ‘condition’ (*F*_(1,81.22)_ = 6.46, *p* = .013), with slower responses in incongruent compared to congruent trials, and a significant main effect of ‘phase’ (*F*_(1,82.40)_ = 79.15, *p* < .001), reflecting overall faster responses in the second session. No other effects or interactions were significant (all *p*-values > .42).

Levene’s tests indicated no significant differences in variance between groups or phases for either WM condition (all *p*-values > .09). Quantile regression analyses of change scores revealed no group differences at lower or median quantiles (τ = .25–.50, all 95% CIs including 0). However, a significant negative effect of SI emerged at the upper quartile (τ = .75: β = −0.05, 95% CI [−0.087, −0.025]), indicating that the largest practice-related gains observed in controls were attenuated in SI. Complete results and graphical displays are reported in Supplementary Material 2.

### 3.2. Inhibitory control

Accuracy analyses on the congruent condition revealed no significant main effects or interactions (all *p*-values > .11). In the incongruent condition, there was a non-significant effect of ‘phase’ (*F*_(1,28)_ = 3.29, *p* = .08), alongside a non-significant effect of ‘group’ and a non-significant ‘phase-by-group’ interaction (both *p*-values > .83) (Figure 3B, top inset). Response time analyses on the congruent condition showed a non-significant effect of ‘phase’ (*F*_(1, 28)_ = 3.47, *p* = .08), together with a non-significant effect of ‘group’ and a non-significant ‘phase-by-group’ interaction (both *p*-values > .66). The incongruent condition revealed no significant effect (all *p*-values > .30) (Figure 3B, bottom inset). In the integrated model, with ‘condition’ (congruent, incongruent) as a fixed effect, we found a significant main effect of phase (*F*_(1, 84.68)_ = 8.13, *p* = .005), indicating that participants were more accurate in the second session. There were no other significant effects or interactions, including the three-way interaction (‘condition’ × ‘phase’ × ‘group’: *p* = .98). For response time, the model revealed a significant main effect of condition (*F*_(1, 81.22)_ = 6.46, *p* = .013), with slower responses in incongruent compared to congruent trials, and a significant main effect of phase (*F*_(1, 82.40)_ = 79.15, *p* < .001), reflecting overall faster responses in the second session. No other effects or interactions were significant (all *p*-values > .42).

### 3.3. Context-free vocabulary navigation

Semantic fluency results (Figure 3C, top inset) revealed significant main effects of ‘language’ (*F*_(1,126)_ = 191.56, *p* < .001) and ‘phase’ (*F*_(1,126)_ = 7.13, *p* = .009), the latter revealing a significant increase in semantic fluency from the pre- to the post-exertion phase. Phonemic fluency outcomes (Figure 3C, bottom inset) showed a significant main effect of ‘phase’ (*F*_(1,126)_ = 9.13, *p* = .003), typified by a significant increase in phonemic fluency performance from the pre- to the post-exertion phase. No other main or interaction effects reached significance in either fluency task (all *p*-values > .24).

## 4. Discussion

This study investigated the impact of SI on WM via a cognitive exertion paradigm. We found that SI, as opposed to TC, prevented practice-driven boosting of integration–recall outcomes, even adjusting for key aspects of bilingual experience. No such effects were observed on inhibitory and fluency performance. These results shed light on the cognitive demands of SI as a distinct form of bilingual processing.

Prior works have shown that WM outcomes are associated with SI experience (García et al., 2019) as well as interpreting quality (Bae & Jeong 2021; Injoque-Ricle et al., 2015; Mellinger & Hanson, 2019), and relevant electrophysiological signatures are distinctly modulated during SI (Boos, 2022). Our pre/post study extends such findings, indicating that WM resources are markedly affected after 10 minutes of this activity.

Crucially, post-task WM performance differed between groups. Integration–recall outcomes improved after TC. This likely reflects well-established practice and repetition effects in WM tasks, manifested as measurable gains through increased familiarity, greater encoding efficiency or more strategic resource allocation (Chein & Morrison, 2010; Jonides et al., 2008). Conversely, no such improvement emerged after SI, suggesting that this task hindered the consolidation or reactivation of learning-related mechanisms. This aligns with resource depletion models, which posit that high-load tasks temporarily exhaust executive resources, impairing subsequent performance or learning (Chen & Kalyuga, 2020; Just & Carpenter, 1992).

In the present design, the selective absence of WM gains after SI could reflect a combination of (1) acute resource depletion during SI and (2) interference with consolidation processes due to the multitasking demands of interpreting (Christoffels et al., 2006). Additionally, the greater variability observed in WM performance within the SI group may reflect inter-individual differences in cognitive resilience or interpreting strategies, further supporting the view that SI imposes heterogeneous cognitive loads that differentially affect WM outcomes (Dong et al., 2018). Importantly, these group differences were not explained by increased variability: variance did not differ between groups or phases, and quantile analyses indicated that only the control group showed practice-related gains at the upper tail of the integration–recall distribution. Thus, SI did not enhance heterogeneity per se, but rather prevented the emergence of high-gain learners typically observed after practice.

Again, such a disruption emerged only in the integration–recall condition, which, unlike the recall-only condition, requires binding different processes (viz., shape and color recognition) (Baddeley et al., 2011; Birba et al., 2017; Brockmole et al., 2008). Suggestively, and as corroborated in the TC group, integration–recall mechanisms are more susceptible to training and interference effects than recall-only mechanisms (Allen et al., 2006; Parra et al., 2009) – a finding that highlights the selectivity of SI-related WM effects for high-load conditions. Compatibly, cognitive advantages associated with sustained SI practice are systematic for WM tasks that involve concurrent cognitive operations but inconsistent for tasks that tap on unimodal retrieval (García et al., 2019). For example, interpreters show training effects in WM tasks involving both audio and visual stimuli, but not under a single-modality condition (Morales et al., 2015). This selectivity might reflect the critical role of resource management and task scheduling during SI, two aspects that are distinctly taxed during multimodal WM processes (Mizuno, 2005).

The above pattern survived covariation with age of L2 appropriation, years of L2 use, L2 proficiency and SI competence. This is noteworthy, as WM performance in bilinguals is often influenced by such factors (Köpke & Nespoulous, 2006; Lukasik et al., 2018; Miyake & Friedman, 2013; Tzou et al., 2012). Thus, contrary to other WM dynamics in this population, SI-driven WM exertion seems consistent beyond inter-individual variability in bilingual experience. Moreover, such WM exertion effect was not observed in the TC group, mirroring evidence that other SI-related effects on executive mechanisms are absent in single-language tasks, like text listening and shadowing (Boos, 2022; Hervais-Adelman et al., 2011, 2015; Hyönä et al., 1995). Since the WM task was identical (in stimuli, structure and duration) for both groups, the observed draining of WM resources would be a differential consequence of SI rather than a manifestation of sustained discourse processing (or prior WM taxing) at large.

Furthermore, exertion effects during L1–L2 SI seem distinctly operative in WM functions, as they were not observed on inhibitory or fluency outcomes. Similarly, while SI experience is consistently associated with WM advantages, no systematic enhancement is observed on either of those domains (Aparicio et al., 2017; García et al., 2019; Morales et al., 2015; Stavrakaki et al., 2012; Van de Putte et al., 2018). For example, despite inducing neuroanatomical changes, SI training yielded null effects on a Simon task – a gold-standard measure of response inhibition (Van de Putte et al., 2018). As proposed elsewhere (García et al., 2019), this might be so because concomitant processes during SI (e.g., source-language comprehension and target-language production) need to be active in parallel rather than alternately suppressed. Still, null effects on inhibition and fluency may be partly driven by our design. Indeed, both tests, unlike the WM task, had demands other than SI between their pre- and post-exertion versions. Though further research is needed to rule out this confound, our findings suggest that SI does not uniformly impact domain-general processes at large.

Incidentally, fluency results revealed better outcomes in L1 (Chinese) than in L2 (English). This pattern might reflect two phenomena. First, in Asian languages, animal fluency outcomes evince disproportionately high rates of zodiac animals, a list that is rotely learned and quickly produced across L1 speakers (Sung et al., 2025). Second, word retrieval outcomes are generally lower in L2 than in L1, likely due to less entrenchment in the former (French & Jacquet, 2004) – a difference that may override immediate L1–L2 priming effects. However, fluency results might be influenced by the fixed administration of the L1 before L2 condition, inviting further research with strategic manipulations.

Our study carries theoretical implications. Links between SI and WM are acknowledged in different accounts, but mainly supported by cross-sectional studies (García et al., 2019). Rooted in a pre/post design, our results suggest that SI may be causally involved in the exhaustion of WM resources, offering mechanistic constraints for cognitive modeling. More particularly, our findings support the view that SI effects are typified by ‘demand-based domain-specificity’ (García et al., 2019: 736). While this claim was first advanced by reference to SI-induced benefits (García, 2014), the abolition of practice-related WM effects shows that even SI-induced disadvantages are also confined to functions distinctly recruited during the task. This extends the interpreter advantage hypothesis, suggesting that the selective impact of SI on its central domains may manifest not only as long-term gains but also as immediate decline. Accordingly, accounts of SI-related effects could be fruitfully expanded by detailing how immediate resource depletion progressively leads to diachronic boosts – e.g., through entrenchment or activation threshold adaptations.

This work is not without limitations. First, though adequately powered and similar to that of previous works (e.g., Babcock & Vallesi, 2017; Becker et al., 2016; Čeňková et al., 2014; Tzou et al., 2012), our sample size was modest. As in previous bilingualism studies (e.g., Bialystok et al., 2004; Calabria et al., 2012; Blanco-Elorrieta & Pylkkänen, 2017; Declerck et al., 2015), this was mitigated via linear mixed-effects models with a simple structure and within-subject data, which enhances statistical sensitivity by reducing inter-individual variability (Charness et al., 2012). Nonetheless, future studies should aim to replicate these findings with larger and more diverse samples. Second, the sample consisted solely of interpreting students. Given that cognitive outcomes may vary widely between trainees and professionals (García et al., 2019), future works should test whether similar exertion effects occur across both populations. Third, L2 proficiency and SI competence levels were based solely on self-report data. Though widely used (Hulstijn, 2012) and predictive of objective language outcomes (Langdon et al., 2005; Marian et al., 2007; Gollan et al., 2012), these could be prone to self-image and desirability biases. Future works should complement such measures with formal test results as inclusion criteria. Fourth, participants received no topic information prior to SI. While this mirrors previous works (Bae & Jeong, 2021; Boos et al. 2022; Tzou et al. 2012), frequent professional scenarios (Chang et al. 2018) and participants’ experience during formal training, it deviates from standard, recommended practice, inviting studies on how pre-task briefing might influence WM demands.

Fifth, while our WM task has been explicitly used to operationalize recall skills (Brockmole et al., 2008), these are variously intertwined with recognition abilities, especially in the present paradigm. Future works could tackle this issue through tasks that more clearly demarcate both domains, disentangling the possible contributions of recognition skills to present results. Sixth, although the use of colors as stimuli minimizes semantic associations, their nameability raises the possibility of verbal coding strategies (Baddeley, 2003). While our data do not suggest that participants systematically engaged in such strategies, this possibility cannot be fully excluded, and thus, present effects should be interpreted as reflecting domain-general, multimodal and possibly language-linked mechanisms. Seventh, the SI session may have been too short for exertion effects to manifest in the post-SI inhibitory and fluency measures. Future works could examine whether stronger or longer depletion influences tasks administered later in the protocol. Eighth, inhibition and fluency tasks were administered after the post-training WM task. As such, their underlying resources may have been depleted but then replenished by the time of testing. While the short delay (20 minutes) and the added fatigue from the WM task make full recovery unlikely, future studies should counterbalance tasks to more firmly assess the selectivity of observed WM effects. Alternatively, SI-induced exertion effects may not have been strong enough to persist until the inhibitory and fluency tasks were performed. Further studies could assess this possibility with a prolonged SI session. Ninth, while our pre/post design offers an advantage over purely cross-sectional approaches by capturing within-subject change (Charness et al. 2012), our main finding – the interaction between group and phase – relies on a between-subjects comparison. Observed differences, then, could be partly influenced by confounds beyond our design. Within-subject or crossover designs would help to more precisely isolate the phenomena targeted herein.

Finally, our SI task involved the L1–L2 direction only. Though widespread in the Chinese context, this direction involves neurocognitive demands that differ from those of L2–L1 interpreting (Pérez et al., 2022; Rinne et al., 2000; Zheng et al., 2020), as evidenced by distinct behavioral performance and greater activation along inferior frontal regions implicated in WM and linguistic processes (García, 2019; Rinne et al., 2000; Tommola et al., 2000). Higher demands for the L1–L2 direction have also been reported during sentence and word translation, manifested as increased temporo-frontoparietal connectivity (Zheng, et al., 2020) and higher frontal theta power (Pérez et al., 2022), respectively. Therefore, present results should be taken to reflect L1–L2 SI demands in particular, calling for further studies on how directionality modulates WM exertion.

## 5. Conclusion

In sum, our study shows that a brief period of SI interferes with practice-driven boosting of WM integration–recall outcomes. This effect seems specific to both SI (relative to single-language discourse processing) and WM (as opposed to other cognitive domains). Future works along this line can further illuminate the interplay between cross-linguistic tasks and the cognitive mechanisms that support performance in bilingual populations.

## Supporting information

10.1017/S1366728925100898.sm001 Chou et al. supplementary material Chou et al. supplementary material

## Data Availability

All experimental data and scripts are available via the Open Science Framework at https://osf.io/p37wr/?view_only=73e68d4e0e254400a611f9cb94d06c90.

## Footnotes

1 Proposed by Paradis (2009), the term ‘appropriation’ is a hypernym encompassing both incidental acquisition (which typically occurs before age 7) and metalinguistic learning (more common after age 7). As in previous works (Kogan et al., 2024), this concept is relevant to our design given that we recruited both early and late bilinguals, with no constraints on how they developed their L2 skills.

## Supplementary material

The supplementary material for this article can be found at http://doi.org/10.1017/S1366728925100898.
