# Supplementary material for: Working memory exertion after simultaneous interpreting in bilinguals
Source: Biling (Camb Engl). 2026 Jan 2:1–11. Online ahead of print. doi: 10.1017/S1366728925100898 (PMC12885050; doi:10.1017/S1366728925100898)
Supplement: Chou et al. supplementary material [file S1366728925100898sup001.pdf]

Supplementary material

1. Power estimation

Statistical power estimation was conducted by using the simulate function from the lmer package (Bates et al., 2015) in R (Team R, 2021), following methods from prior research (Vitale et al., 2023) including studies on bilingualism (Fu et al., 2024). We generated 1,000 simulated datasets, each containing *n* participants, based on the main mixed-effects model's structure (accuracy ~ phase * group + (1 | subject)), which included 'phase and 'group' as fixed effects and 'Participant' as a random intercept. In each simulated dataset, a random subset of trials was marked as missing and excluded to mirror the preprocessing steps applied to the empirical data. Separate simulations were performed by progressively increasing the effect size of the two-way interaction ('phase' × 'group') to evaluate power under varying conditions. The proportion of models in which the two-way interaction was detected at $p < .05$ served as the power estimate. For simulations with $n = 24$ participants, the model consistently identified a significant interaction in all 1,000 runs, yielding a power estimate of 1, indicating that in all 1,000 runs, the model consistently identified a significant two-way interaction.

2. Variability analyses

To test whether between-group differences were explained by variability in WM performance, we first conducted Levene's tests. For the integration-recall condition, no significant variance differences were observed between SI and TC in either the pre-exertion phase ($F_{(1,47)} = 2.57$, $p = .116$) or the post-exertion phase ($F_{(1,46)} = 2.93$, $p = .094$). Likewise, no differences emerged between the pre- and the post-exertion phase within the SI group ($F_{(1,48)} = 0.04$, $p = .837$) or within the TC group ($F_{(1,45)} = 0.02$, $p = .890$). For the recall-only condition, no significant variance differences were found between SI and TC in either the pre-exertion phase ($F_{(1,48)} = 1.50$, $p = .226$) or the post-exertion phase ($F_{(1,48)} = 1.66$, $p = .204$). Similarly, comparisons between phases revealed no differences within the SI group ($F_{(1,50)} = 0.22$, $p = .641$) or within the TC group ($F_{(1,46)} = 0.37$, $p = .545$). These results indicate that group and phase differences in WM accuracy were not driven by differences in variance.

To further explore distributional patterns, we computed individual change scores (subtraction between the post- and pre-exertion phase) and conducted quantile regression analyses. In the recall-only condition, no group effects were found at lower, median, or upper quantiles ($\tau = .25$: $\beta = 0.00$, 95% CI [−0.066, 0.051]; $\tau = .50$: $\beta = -0.02$, 95% CI [−0.070, 0.049]; $\tau = .75$: $\beta = -0.01$, 95% CI [−0.021, 0.034]), indicating that SI and TC participants

showed comparable changes across the distribution. In the integration-recall condition, no group differences emerged at τ = .25 (β = −0.01, 95% CI [−0.065, 0.018]) or τ = .50 (β = −0.04, 95% CI [−0.078, 0.018]). However, a significant negative effect of SI appeared at the upper quartile (τ = .75: β = −0.05, 95% CI [−0.078, −0.015]), indicating that the largest practice-related gains observed in the TC were attenuated in SI.

As illustrated in Figure S1, the TC group displayed a clear rightward tail in the distribution of change scores for the integration-recall task, reflecting high improvers, whereas such gains were absent in SI. Together, these findings clarify that SI did not increase overall variability, but rather prevented the emergence of strong practice-related improvements in WM binding.

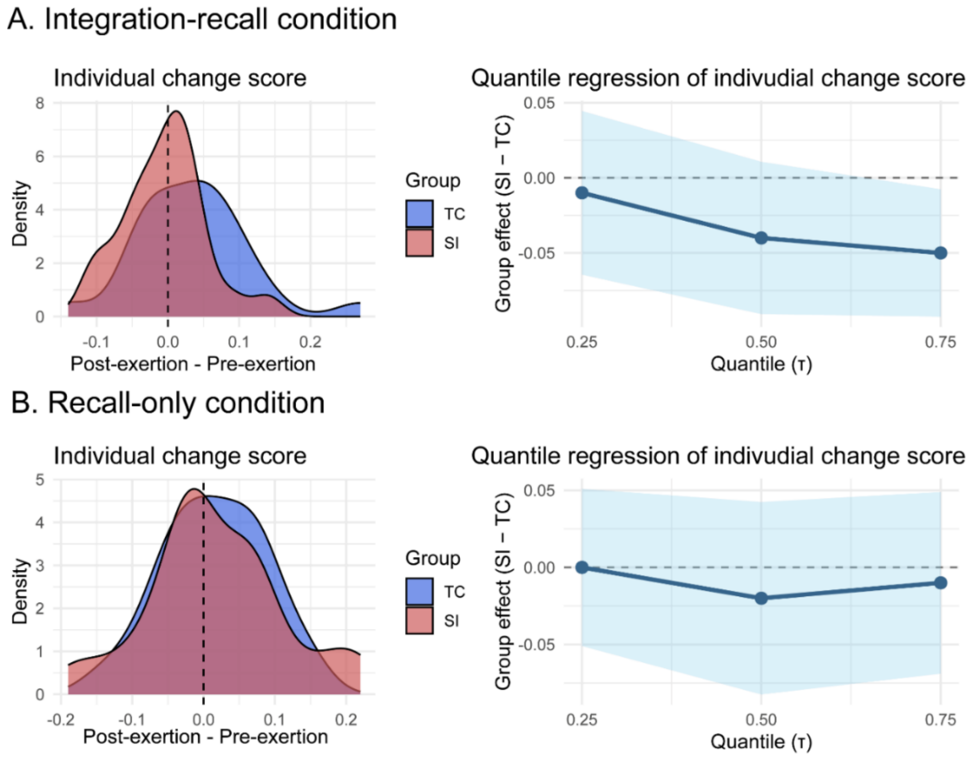

Figure S1. Distributional analyses of individual change scores (subtraction between the post- and the pre-exertion phase) and quantile regressions. (A) Integration-recall condition. Left: Kernel density plots showing the distribution of individual change scores for the text comprehension (TC) and simultaneous interpreting (SI) groups. Right: Quantile regression estimates of group differences (SI − CT) across quartiles (τ = .25, .50, .75), with 95% bootstrap confidence intervals. The TC group displayed a rightward tail, indicating practice-related improvements, whereas these gains were absent in SI. (B) Recall-only condition. Left: Kernel density plots of individual change scores by group. Right: Quantile regression estimates showed no significant group differences across quartiles.

References

Bates D, Maechler M, Bolker B, et al. Package ‘lme4’. 2015;12(1):2.

Fu Y, Bermúdez-Margaretto B, Beltrán D, Huili W, Dominguez AJSiSLA. Language proficiency modulates L2 orthographic learning mechanism: Evidence from event-related brain potentials in overt naming. 2024;46(1):119-140.

Team R. R: A language and environment for statistical computing (R Foundation for Statistical Computing;).[Google Scholar]. 2021.

Vitale F, Urrutia M, Avenanti A, De Vega MJSC, Neuroscience A. You are fired! Exclusion words induce corticospinal modulations associated with vicarious pain. 2023;18(1):nsad033.
